# Identification of a likely pathogenic structural variation in the *LAMA1* gene by Bionano optical mapping

**DOI:** 10.1038/s41525-020-0138-z

**Published:** 2020-08-12

**Authors:** Min Chen, Min Zhang, Yeqing Qian, Yanmei Yang, Yixi Sun, Bei Liu, Liya Wang, Minyue Dong

**Affiliations:** 1 Women’s Hospital, School of Medicine, Zhejiang University, 1, Xueshi Road, Hangzhou, Zhejiang 310006 People’s Republic of China; 2 Key Laboratory of Reproductive Genetics (Zhejiang University), Ministry of Education, 1, Xueshi Road, Hangzhou, Zhejiang 310006 People’s Republic of China; 3 Key Laboratory of Women’s Reproductive Health of Zhejiang Province, 1, Xueshi Road, Hangzhou, Zhejiang 310006 People’s Republic of China; 4 Hangzhou Women’s Hospital, 369, Kunpeng Road, Hangzhou, Zhejiang 310002 People’s Republic of China

**Keywords:** Genetics research, Diagnosis

## Abstract

Recent advances in Bionano optical mapping (BOM) provide a great insight into the determination of structural variants (SVs), but its utility in identification of clinical likely pathogenic variants needs to be further demonstrated and proved. In a family with two consecutive pregnancies affected with ventriculomegaly, a splicing likely pathogenic variant at the *LAMA1* locus (NM_005559: c. 4663 + 1 G > C) inherited from the father was identified in the proband by whole-exome sequencing, and no other pathogenic variant associated with the clinical phenotypes was detected. SV analysis by BOM revealed an ~48 kb duplication at the *LAMA1* locus in the maternal sample. Real-time quantitative PCR and Sanger sequencing further confirmed the duplication as c.859-153_4806 + 910dup. Based on these variants, we hypothesize that the fetuses have Poretti-Boltshauser syndrome (PBS) presenting with ventriculomegaly. With the ability to determine single nucleotide variants and SVs, the strategy adopted here might be useful to detect cases missed by current routine screening methods. In addition, our study may broaden the phenotypic spectrum of fetuses with PBS.

## Introduction

Accurate molecular diagnosis of genetic disorders is crucial to families with history of diseases and couples with consecutive abnormal fetuses showing similar phenotypes. The identification of pathogenic variants has a great implication for disease diagnosis, treatments, and reproduction guidance. In the past few years, whole-exome sequencing (WES) has greatly facilitated the identification of candidate disease-causing variants in coding regions^1,2^. Based on the next-generation sequencing, WES is capable of identifying single nucleotide variants (SNVs) and small variants. Variants in genetic disorders are constantly discovered, yet ~60% of cases remain undiagnosed^3,4^. One reason is that some structural variants (SVs) are challenging to detect using short reads-based technologies^5^. SVs are known to be implicated in a wide range of genetic diseases^6^, including copy number variants (CNVs), inversions, translocations, and so on. Currently, chromosome microarray (CMA) remains the most common method to determine CNVs^7^. However, it is not able to identify small or balanced SVs, nor does it provide information about the location and orientation^8^. Hence, when common molecular tests cannot identify any potential pathogenic variants for families with a clear indication of genetic problems, new techniques are required to fill the gaps.

Advances in genome mapping technologies continue to provide great insights into the determination of SVs^9,10^. On the basis of nanochannel-based genome mapping technology, Bionano optical mapping (BOM) utilizes enzymes to label high molecular weight DNA without breaking the DNA or polymerase chain reaction (PCR), and fluorescently tagged mega base size DNA are obtained^11^. These long fragments of DNA are linearized through pillars and imaged in nanochannels. A de novo genome assembly is derived from many copies of a native DNA molecule, which allows BOM to detect CNVs, translocations and inversions, as well as other complex SVs^10,12^. It also offers the opportunity to understand the size, location, and orientation of SVs. So far, BOM has been successfully applied in cancer studies^13^, and Duchenne and Becker muscular dystrophy diagnosis^14^, but its utility in determination of clinical likely pathogenic variants need to be further demonstrated and proved.

Poretti-Boltshauser syndrome (PBS; MIM: 615960)^15,16^ is an autosomal recessive disorder characterized by cerebellar dysplasia, cerebellar cysts, and abnormally sized fourth ventricle. Most patients present with ataxia, language delay, cognitive impairment, developmental delay, and ocular motor apraxia. High myopia, abnormal eye movements, and retinal dystrophy are also seen in some patients. PBS is associated with biallelic pathogenic variants in the *LAMA1* gene located on 18p11.31. This gene encodes one of the alpha 1 subunits of laminin, which plays an essential role in many biological processes, involving cell adhesion, differentiation, migration, signaling, neurite outgrowth, and metastasis^17^. Besides SNVs, three multi-exon deletions^16,18,19^ and a gross insertion including the entire gene^20^ have been reported in patients with PBS. However, some potential disease-causing SVs may be missed by using WES and CMA.

In this study, we identified two likely pathogenic variants at the *LAMA1* locus in a family with two consecutive pregnancies affected with ventriculomegaly by using BOM together with WES, CMA, and Sanger sequencing. Based on these variants, we hypothesize that the fetuses are affected with PBS. With the ability to determine SNVs and SVs, the strategy adopted here might be useful to detect cases missed by current routine screening methods. Our data further demonstrated the clinical utility of BOM in genetic disorders.

## Results

### Molecular characterizations

**Fig. 1:**
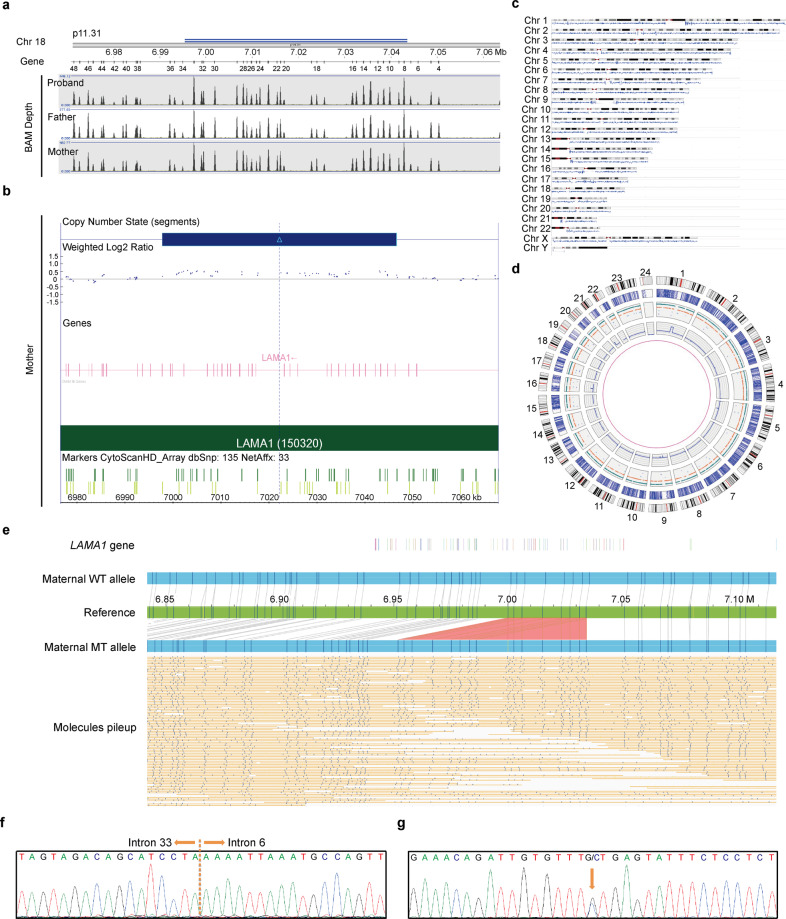
Characterization of disease-causing variants at the *LAMA1* locus. **a** Coverage based on WES Bam depth implied a possible gain of partial *LAMA1* gene in the proband and mother. The height of peaks indicates the number of reads. **b** A suspected gain encompassing exons 6–33 was observed by CMA in the mother with lowered cutoff value (gains ≥ 25 kb and losses ≥ 25 kb). **c** Genome coverage of de novo assembly generated from 389 consensus genome maps on hg19 by using BOM. Reference genome is plotted by G banding patterns, in which centromeric bands and stalks are marked in red, while variable heterochromatic region in black. Horizontal blue shading indicates regions aligned with individual long DNA molecules. **d** Circos plot depicting genomic variations. The tracks from outer to inner circles are chromosome cytobands, canonical gene, insertions (green), deletions (orange), inversions (blue), duplications (purple), and copy number variants. No translocation was found in the sample. **e** BOM revealed a heterozygous multi-exon duplication in the mother. Single-molecule maps (orange horizontal lines) assembled de novo genome maps (blue bars) are aligned to the reference (green bar). The location of *LAMA1* exons are shown as colorful vertical lines at the top. The wild-type (WT) allele can be seen above the reference, while the mutant (MT) allele is shown below. Nicking sites are displayed as black vertical lines and blue dots. The gray lines indicate the alignment between the reference and assembled map. The light red area indicates the duplication where two copies of reference endonuclease sites are presented in the MT allele. **f** Sanger sequencing showing the duplication (NM_005559: c.859-153_4806 + 910dup) in the mother. The breakpoint junction is indicated by yellow dash line. **g** Sanger sequencing showing the splicing variant (NM_005559: c. 4663 + 1 G > C) in the father. The arrow indicates the mutant variant.

WES was carried out on the second fetal and parental DNA samples, and a splicing pathogenic variant at the *LAMA1* locus (NM_005559: c. 4663 + 1 G > C) inherited from the father was detected in the proband. No other pathogenic variant associated with the clinical phenotypes was identified. Interestingly, the splicing variant was present in 27.3% of all reads covering this position in the proband and 46.3% in the father, indicating mosaicism or an extra copy of this region in the proband. Given that SVs might be causal variants of PBS, we speculated that there might be another pathogenic variant on the other allele that is beyond the detection ability of WES. By further reviewing the WES data, we noticed a slightly increased coverage of exon 8–32 at the *LAMA1* locus in the proband’s and maternal samples (Fig. 1a), indicating a possible duplication *in trans* in the proband. However, the WES signals of this duplication was equivocal. As there was no fetal DNA sample left after WES, subsequent studies were carried out on parental samples. To determine whether there was an SV, CMA was performed on the maternal sample. No significant abnormality was identified by CMA. Notably, when we lowered the cutoff value (gains ≥ 25 kb and losses ≥ 25 kb) and looked into the *LAMA1* gene, a suspected gain encompassing exons 6–33 was observed (Fig. 1b). At this point, both WES and CMA implied a possible and undetectable duplication in the *LAMA1* gene. In this case, it would be questionable whether this duplication really exists; if so, where it is and how it duplicates. To solve these questions, other technologies such as BOM with the capability of detecting long DNA molecules would be necessary.

### BOM and SV detection

In attempt to confirm and characterize the SV, BOM was performed on the maternal sample. An average mapped coverage depth of 157× was achieved. A total of 2,076,484 molecules longer than 150 kb were captured and a de novo genome map assembly was created from 389 consensus genome maps (Fig. 1c, Table 1). The average consensus genome map is 11.338 Mb in length. More than 85% of the genome maps could be aligned to the human reference hg19, achieving a breadth of coverage of 91.8%. A total of 4280 clustered SVs relative to hg19 were detected (Fig. 1d, Table 1). The density of labels was ~15.14 times per 100 kilo base pairs (kb) on average.

**Table 1:** Genome mapping summary.

Molecule summary (filtered):	
Total number of molecules > 150 kb	2,076,484
Total length (Mb)	612,482.829
Average length (kb)	294.961
Molecule N50 (kb)	309.208
Average label density (/100 kb)	15.140
Coverage of the reference (hg19)	197.851
Molecules aligned to the reference:	
Total number of molecules aligned	1,865,391
Fraction of molecules aligned	0.898
Effective coverage of the reference (hg19)	157.011
Average confidence	36.8
De novo assembly:	
Diploid genome map count	389
Diploid genome map length (Mb)	5852.162
Diploid genome map N50 (Mb)	70.368
Haploid genome map count	262
Haploid genome map length (Mb)	2970.517
Haploid genome map N50 (Mb)	70.338
Total reference length (Mb)	3095.677
Total number of genome maps aligned (fraction)	331 (0.85)
Total unique aligned length (Mb)	2841.934
Total unique aligned length/reference length	0.918
Molecules aligned to the assembly:	
Total number of molecules aligned	1,859,320
Fraction of molecules aligned	0.895
Effective coverage of assembly	96.548
Average confidence	47.7
Structural variation summary:	
Deletions	1336
Insertions	2801
Duplications	52
Inversion breakpoints	91

SV analysis revealed an ~48 kb heterozygous duplication at the *LAMA1* locus (Fig. 1e). More importantly, high-resolution fluorescence imaging of linearized DNA passing through nanochannels directly shed light on the duplication as a tandem duplication, i.e., the duplication was present adjacent to the 3' of the original copy in the same orientation at the *LAMA1* locus. According to the fluorescent labels, the 5' breakpoint was located within exons 6–15, while the 3' breakpoint within exons 30–37. BOM provided a remarkable insight into the presence of an intrachromosomal duplication.

### Confirmation of variants via RT-qPCR and/or Sanger sequencing

To validate the duplication and define the breakpoints at single nucleotide level, we performed real-time quantitative PCR (RT-qPCR) and Sanger sequencing on the maternal sample. RT-qPCR revealed two copies of exons 6 and 34, and three copies of exons 7 and 33 (data not shown). Figure 1f displayed the amplification of the junction region, where part of intron 33 was followed by part of intron 6 in the same orientation. Sanger sequencing confirmed the carrier status of the mother, and further demonstrated the breakpoints in intron 6 and intron 33. Thus, the duplication was reported as c.859-153_4806 + 910dup, encompassing 48,161 base pairs (bps). Considering the WES data, the mother transmitted the duplicated allele to the proband. The splicing variant was also confirmed by Sanger sequencing. As shown in Fig. 1g, the father carried the splicing variant at the *LAMA1* locus (NM_005559: c. 4663 + 1 G > C).

### Variants interpretation

Genetic analysis described above revealed that the proband carried compound heterozygous variants of c.859-153_4806 + 910dup and c. 4663 + 1 G > C in the *LAMA1* gene. The variant c.859-153_4806 + 910dup inherited from the mother has not been reported. It spanned from exons 7 to 33, which encoded several Laminin EGF-like domains and partial domain I. Moreover, it was absent from the controls in Exome Aggregation Consortium (ExAC) database. The multi-exon duplication was predicted to affect the domain I, which is thought to interact with other laminin chains to form a coiled-coil structure and thus destroy the protein structure. There was no reported case in the DECIPHER database involving the duplicated region. Therefore, this duplication was classified as likely pathogenic. The variant c. 4663 + 1 G > C transmitted from the father had been reported in a patient with PBS previously^18^. It was predicted to cause a splice defect at the mRNA level and was not found in the ExAC database. Thus, it was also classified as likely pathogenic.

## Discussion

For the past few years, diagnoses of genetic disorders mainly focus on the identification of SNVs by WES and CNVs by CMA, but a large portion of cases remain undiagnosed^21^. It is probably due to the inabilities of such platforms to characterize certain kinds of SVs^22^, which represent an important class of genetic variations and are usually causative for many genetic disorders^23^. In our study, these conventional platforms could neither provide a definite determination of the duplication nor reveal the location and orientation. Moreover, WES screens only exons, leaving 99% genome undetected. In addition, short-read sequences are typically aligned to a reference sequence that limits the identification of large SVs. Furthermore, SVs might be missed when references are incorrect or incomplete. Recent advances in single-molecule sequencing and whole-genome mapping are showing promising utility in comprehensive structural variation analysis, particularly for duplications, insertions, inversions, and translocations^24^. They are able to produce long-read-based de novo assemblies with remarkable quality and are capable of reconstructing the entire human genome, which is impossible by short-read sequencing. However, single-molecule sequencing technologies, such as Pacific Biosciences and Oxford Nanopore Technologies are either too expensive to obtain sufficient coverage, or suffered from poor accuracy^25^. Taking the cost, time, and throughput into account, whole-genome mapping-based BOM may be a good alternative for SVs detection. Although it is unable to offer base-level sequence information, its abilities to visualize DNA structure and to reveal a wide spectrum of SVs make it a good complement to short-read sequencing^26^. Since the resolution of breakpoints is limited to label density, more enzymes can be used to improve the performance of SVs characterization. Alternatively, as shown in our study, the combination with other technologies could provide more comprehensive understandings of the SVs. In addition, the consistent results from different methods ensure the accurate determination of variants.

Here, we successfully identified one duplication (c.859-153_4806 + 910dup) and one splicing variant (c. 4663 + 1 G > C) at the *LAMA1* locus in a family with two consecutive pregnancies affected with ventriculomegaly. Mild ventriculomegaly has been reported in three children with PBS^16,18^. Based on the biallelic-predicted pathogenic *LAMA1* variants, we hypothesize that the fetuses have PBS presenting with ventriculomegaly. As far as we know, all the cases with PBS described in the literatures are children and adults^16,18,19,27–29^. We reported the characterizations presented in the second trimester. The different phenotypes are likely caused by the variants’ types and locations. Our study showed that the onset of PBS might be as early as the gestation period. These findings may broaden the phenotypic spectrum of fetuses with PBS, but additional studies are needed to better understand the fetal phenotypes. It should be noted that ventriculomegaly, if present, is nonspecific for PBS. However, it highlights that attention should be given to the patients with ventriculomegaly and their *LAMA1* gene could be screened. Specific reproduction advices can then be offered to the couple based on the results.

In this study, a strategy involving BOM, WES, CMA, RT-qPCR, and Sanger sequencing for disease-causing variants detection was employed. It might be useful for cases missed by current routine screening methods. We successfully applied it to a family with consecutive abnormal pregnancies and accurately identified two likely pathogenic variants in the *LAMA1* gene. BOM further characterized the location and orientation of the SV, which could not be revealed by WES or CMA, proving its values in determination of likely pathogenic SVs.

## Methods

### Patients and biological samples

A 29-year-old healthy woman who had two consecutive pregnancies with ventriculomegaly was referred to the Department of Reproductive Genetics, Women’s Hospital, School of Medicine, Zhejiang University, Zhejiang, China. Ultrasound scans showed dilated bilateral ventricles, dilated third ventricle, and aqueductal stenosis at the 17th week of gestation for the first fetus. For the second fetus, MRI scan indicated dilated bilateral ventricles at the 22th gestational week, and ultrasound scans showed dilated bilateral ventricles and brain parenchyma compression at the 31th week. For both fetuses, no other abnormalities were observed and nuchal translucency thickness were normal. Their CMV results and toxoplasmosis test results were both negative. Cell-free fetal DNA tests also showed negative results. Both pregnancies were terminated. Autopsy was not performed on either fetus. This family has no other conditions or affected family members, and the couple are not consanguineous. Peripheral blood samples from the couple were obtained. Genomic DNA samples were extracted with QIAamp® DNA Blood Mini Kit (QIAGEN, Germany) according to the manufacturer’s protocol. In addition, fetal DNA sample from the second fetus was collected for WES test. Signed informed consent was obtained from the couple. This study was approved by the Ethics Committee at Women’s Hospital, School of Medicine, Zhejiang University. This study is compliant with the ‘Guidance of the Ministry of Science and Technology for the Review and Approval of Human Genetic Resources’, which requires formal approval for the export of human genetic material or data from China.

### Whole-exome sequencing

The genomic DNA samples were captured by Agilent SureSelect Human All Exon V5 (Agilent, USA) and sequenced on Illumina HiSeq2000 platform (Illumina, USA). Data were mapped to the hg19 reference human genome using Sentieon BWA (Sentieon, USA). Average sequencing coverage was 90-fold with >95% of the region-of-interest covered at least 20-fold. Variants were filtered through population databases, annotated by Variant Effect Predictor software, and online databases, such as ClinVar, OMIM, and HGMD. CNVs were analyzed by NxClinical 4.1 (BioDiscovery, USA).

### Chromosome microarray

Genotypes and CNVs were detected by Affymetrix CytoScan™ HD array (Affymetrix, USA) following the manufacturer’s instructions. These HD arrays contained ~2,600,000 markers, including 750,000 single nucleotide polymorphic probes and 1,900,000 non-polymorphic probes. Data were visualized and analyzed with the Chromosome Analysis Suite software (Affymetrix, USA) based on hg19 assembly. Gains ≥ 500 kb and losses ≥ 200 kb, containing a minimum of 50 markers in the region were considered as significant.

### Bionano optical mapping

High molecular weight DNA was extracted from whole blood by using Bionano Prep Blood and Cell Culture DNA Isolation Kit (Bionano Genomics, USA). Briefly, blood was subjected to two rounds of red blood cell lysis. After quantitation, white blood cells were immobilized in agarose plugs (Bio-Rad, USA). Plugs were digested with proteinase K (Qiagen, Germany), and washed with wash buffer (Bionano Genomics) and TE buffer (Thermo Fisher Scientific, USA). DNA was recovered, dialyzed, homogenized, and quantitated subsequently. DNA labeling was processed with Bionano Prep™ DLS DNA Labeling kit (Bionano Genomics) according to the kit protocol. In brief, DNA was labeled with DL-Green fluorophores using DLE-1 enzyme at 37 °C for 2 h, digested with proteinase K at 50 °C for 30 min, and cleaned up with 1× DLE-1 buffer. Subsequently, DNA backbone was stained with DNA stain, 5× DTT, and 4× flow buffer for 1 h and homogenized overnight at 4 °C. Labeled DNA was loaded on a Saphyr chip (Bionano Genomics) and run on a Saphyr instrument (Bionano Genomics). The de novo genome map assembly was performed using Bionano Solve™ (Bionano Genomics) and SVs were called against the public Genome Reference Consortium hg19 human assembly. Data were analyzed with Bionano Access™ and Bionano Tools™ on Saphyr Compute Servers (Bionano Genomics).

### RT-qPCR and Sanger sequencing

The newly identified duplication was validated by RT-qPCR. Primers were designed to amplify exons of interest and the β-globin gene was chosen as the reference gene. The reaction was carried out on a LightCycler 480 II system (Roche, Switzerland) in 20 µl volume containing 10 µl of SYBR Premix Ex Taq Polymerase (TaKaRa, Japan), 0.4 µl of each primers (10 µmol/l), and 2 µl of the genomic DNA (20 ng). Each sample was tested in triplicate. An initial denaturation step at 95 °C for 10 s was followed by 40 cycles of 95 °C for 5 s and 58 °C for 30 s, and then a final extension step at 72 °C for 10 min. The dosage ratio of the tested samples was calculated by the ΔΔCt method^30^. Target regions flanking variant sites were amplified (F: 5ʹ-CTGCCTGTCTTTAGGTGCATC-3ʹ and R: 5ʹ-GAGGAAAACTGTACTGACCTTC-3ʹ) and Sanger sequencing was performed on an ABI 3500xL Dx Genetic Analyzer (Applied Biosystems, USA).

### Bioinformatic analysis

CNVs and variants were investigated for their clinical relevance using the following databases: DECIPHER (http://decipher.sanger.ac.uk), OMIM (http://omim.org/), PubMed (http://www.ncbi.nlm.nih.gov/pubmed), Exome Aggregation Consortium (ExAC, http://exac.broadinstitute.org/), ClinVar (https://www.ncbi.nlm.nih.gov/clinvar/), and HGMD (http://www.hgmd.cf.ac.uk/ac/index.php).

### Reporting summary

Further information on experimental design is available in the Nature Research Reporting Summary linked to this article.

## Supplementary Information

Reporting Summary

## Data Availability

The data that support the findings of this study have been deposited in SRA database with the accession code PRJNA636598.
